# Olfactory marker protein regulates prolactin secretion and production by modulating Ca^2+^ and TRH signaling in lactotrophs

**DOI:** 10.1038/s12276-018-0035-z

**Published:** 2018-04-06

**Authors:** Chan Woo Kang, Ye Eon Han, Mi Kyung Lee, Yoon Hee Cho, NaNa Kang, JaeHyung Koo, Cheol Ryong Ku, Eun Jig Lee

**Affiliations:** 1 Brain Korea 21 PLUS Project for Medical Science, Yonsei University, Seoul, Korea; 2 Endocrinology, Institute of Endocrine Research, Yonsei University College of Medicine, Seoul, Korea; 3 Department of Pathology, NHIS Ilsan Hospital, 100 Ilsan-ro Ilsan-donggu, Goyang-si, Gyeonggi-do 10444 Korea; 4 Department of New Biology, DGIST, Daegu, 42988 Korea

## Abstract

Olfactory marker protein (OMP) is a marker of olfactory receptor-mediated chemoreception, even outside the olfactory system. Here, we report that OMP expression in the pituitary gland plays a role in basal and thyrotropin-releasing hormone (TRH)-induced prolactin (PRL) production and secretion. We found that OMP was expressed in human and rodent pituitary glands, especially in PRL-secreting lactotrophs. OMP knockdown in GH4 rat pituitary cells increased PRL production and secretion via extracellular signal-regulated kinase (ERK)1/2 signaling. Real-time PCR analysis and the Ca^2+^ influx assay revealed that OMP was critical for TRH-induced PRL secretion. OMP-knockout mice showed lower fertility than control mice, which was associated with increased basal PRL production via activation of ERK1/2 signaling and reduced TRH-induced PRL secretion. However, both in vitro and in vivo results indicated that OMP was only required for hormone production and secretion because ERK1/2 activation failed to stimulate cell proliferation. Additionally, patients with prolactinoma lacked OMP expression in tumor tissues with hyperactivated ERK1/2 signaling. These findings indicate that OMP plays a role in PRL production and secretion in lactotrophs through the modulation of Ca^2+^ and TRH signaling.

## Reproductive health: Prolactin hormone regulation revealed

Uncovering the regulatory mechanism behind production of the prolactin hormone may help tackle reproductive health problems. As well as triggering milk production in female mammals, prolactin is critical for healthy reproduction in both sexes. An excess of prolactin secreted by cells called lactotrophs in the pituitary gland can cause infertility. While scientists know which hormones stimulate prolactin release, how prolactin levels are regulated is unclear. Eun Jig Lee and Cheol Ryong Ku at Yonsei University in Seoul, Korea, and co-workers demonstrated that the olfactory marker protein (OMP) plays a central role in regulating prolactin production. They found that OMP specifically and highly expressed in lactotrophs. Eliminating OMP expression in mice left a key signalling pathway and calcium ion levels upregulated, resulting in increased prolactin and reduced fertility.

## Introduction

Prolactin (PRL) is a hormone that is mainly secreted by lactotrophs of the anterior pituitary gland and is involved in many biological processes, including reproduction and lactation^1^. The dysregulation of PRL signaling contributes to tumorigenesis—including PRL-secreting adenomas or prolactinomas—leading to pathological hyperprolactinemia. In addition, PRL hypersecretion causes hypogonadism and infertility^2^.

PRL secretion is controlled by multiple factors. Dopamine, secreted by hypothalamic neurons, is the major inhibitor of pituitary PRL secretion^3^, which is induced by thyrotropin-releasing hormone (TRH) and estrogen (E2). TRH is secreted by the hypothalamus and transported to the pituitary gland via circulation to stimulate PRL synthesis and secretion, although the underlying mechanisms are not fully understood^4^.

Olfactory marker protein (OMP) is a small, cytoplasmic protein that is abundantly and almost exclusively expressed in vertebrate olfactory neurons^5–8^. Previous studies have shown that OMP modulates olfactory signal transduction in part by participating in Ca^2^^+^ clearance^8–13^. Microarray and RNA sequencing analyses have revealed that OMP is also expressed in non-olfactory tissues, often with odorant receptors (ORs), which constitute a major class of G protein-coupled receptor (GPCR)^14,15^. However, it is not known whether OMP function is conserved across tissues. Recent studies have suggested a link between OMP and the endocrine system, especially in neuroendocrine neoplasia and hormone secretion^15–18^. However, to date, there have been no studies investigating the role of OMP in the functioning of the pituitary gland, which is considered the master regulator of the neuroendocrine system. To address this issue, the present study investigated OMP expression in the mouse and human pituitary gland, and characterized its mechanism of action in pituitary lactotrophs.

## Materials and methods

### Plasmid constructs and transfection

Plasmids expressing OMP were purchased from Addgene (Cambridge, MA, USA). GH4 cells were seeded at a density of 0.5 × 10^6^ cells/60-mm dish 1 day before transfection. The cells were transfected with the appropriate expression plasmids using the Polyjet transfection reagent (SignaGen, Rockville, MD, USA), and cultured at 37 °C for 24 h, followed by treatment with TRH or saline for an additional 30 min prior to lysis.

### Cell culture

Rat pituitary cell lines, GH3 and GH4, were purchased from the American Type Culture Collection (Manassas, VA, USA) and cultured in Dulbecco’s modified Eagle’s medium supplemented with 10% fetal bovine serum (FBS; Hyclone, Logan, UT, USA) and 1% penicillin/streptomycin (Hyclone). Cells were cultured in a humidified tissue culture incubator at 37 °C in an atmosphere of 5% CO_2_.

### Tissue culture

Pituitary glands were isolated from 20-week old C57BL6 OMP-WT or -KO mice (*n* = 5). The anterior pituitary glands were rapidly removed and processed for explant cultures. All procedures were carried out in a laminar-flow hood. After aseptically trimming adhering tissue residues, the pituitary tissue was transferred to a sterile conical tube and washed with cold HEPES-buffered salt solution (HBSS) buffer. The tissue explants were individually placed on 40-mesh Millicell cell culture inserts (Millipore, Billerica, MA, USA) in 1 ml Ham’s F-10 culture medium supplemented with 10% FBS and antibiotics in plastic culture dishes. The cultures were maintained for up to 1 week under controlled conditions (humidified atmosphere, 37 °C, 5% CO_2_ in air) and the medium was changed daily.

### Quantitative real-time PCR (qRT-PCR) analysis

Total RNA was extracted from GH3 or GH4 cells or mouse pituitary tissue lysates using Isol-RNA lysis reagent (5 PRIME, Hilden, Germany), and complementary DNA was prepared using ReverTra Ace (Toyobo, Osaka, Japan). The following forward and reverse primers were used for amplification: glyceraldehyde 3-phosphate dehydrogenase, 5′-GGATGGAATTGTGAGGGAGA-3′ and 5′-GAGGACCAGGTTGTCTCCTG-3′; PRL, 5′-CATCAATGACTGCCCCACTTC-3′ and 5′-CCAAACTGAGGATCAGGTTCAAA-3′; mouse OMP, 5′-CGTCTACCGCCTCGATTTCA-3′ and 5′-CAGAGGCCTTTAGGTTGGCA-3′; rat OMP, 5′-GCAGTTCGATCACTGGAACG-3′ and 5′-ATCCATGGCATCGGAGTCTTC-3′; and TRHR1, 5′-CATGTTCAATAACGGCCTTTACC-3′ and 5′-GGGCTGGAGAGAAATGAGTTGACA-3′.

### Western blot assay

Whole cell protein lysates were prepared, and the western blot assay was performed according to standard procedures. Briefly, cells were chilled on ice, washed twice with ice-cold phosphate-buffered saline, and lysed in buffer containing 1 mM phenylmethylsulfonyl fluoride and 1× protease inhibitors (Sigma-Aldrich). Protein concentrations were determined with the Bradford assay kit (Bio-Rad, Hercules, CA, USA). Equal amounts of protein in cell lysates were separated by sodium dodecyl sulfate polyacrylamide gel electrophoresis and transferred to a membrane that was incubated with the primary antibody, overnight at 4 °C; the primary antibodies used were rabbit anti-phospho-ERK1/2 (T202/Y204), mouse anti-ERK1/2, and rabbit anti-phospho-PKCα/β (T638/641) (Cell Signaling Tec, MA, USA). Rabbit anti-OMP, goat anti-PRL, and mouse anti-β-actin antibodies were purchased from Santa Cruz Biotechnology (Santa Cruz, CA, USA).

Blots were washed three times with TBST (Tris-buffered saline containing 0.05% Tween 20), and then incubated with horseradish peroxidase (HRP)-conjugated secondary antibody for 1 h at 25 °C. Secondary antibodies were donkey anti-rabbit IgG-HRP antibody (1:5000; Santa Cruz), donkey anti-mouse IgG-HRP antibody (1:5000; Santa Cruz), or donkey anti-goat IgG-HRP antibody (1:5000; Santa Cruz). Immunoreactivity was detected with the SuperSignal West Pico Chemiluminescent Substrate (Thermo Fisher Scientific, MA, USA). The intensity of protein bands was quantified using ImageJ and normalized to that of β-actin in each sample.

### Immunofluorescence analysis

Paraffin-embedded rat pituitary samples were cut into 4 μm sections that were deparaffinized in xylene and rehydrated in a graded series of ethanol. Antigen retrieval was carried out in 10 mM sodium citrate buffer (pH 6.0). Sections were blocked in 5% normal serum for 1 h. Incubation with specific antibodies for rabbit anti-OMP (1:100, Santa Cruz, CA, USA), goat anti-GH (1:200), goat anti-PRL (1:200, Santa Cruz, CA, USA), mouse anti-ACTH (1:200, Santa Cruz, CA, USA), mouse anti-TSH (1:200, Santa Cruz, CA, USA), and mouse anti-FSH (1:200, Santa Cruz, CA, USA) was performed overnight at 4 °C. After washing with TBST, secondary antibodies, donkey anti-rabbit-FITC, donkey anti-goat Cy3, or donkey anti-mouse Cy3 (1:200; Jackson ImmunoResearch, West Grove, PA, USA), were added, and the sections were incubated for 2 h at room temperature. After washing, the sections were mounted with Vectashield medium (Vector Laboratories, Burlingame, CA, USA). Samples were visualized with an Axioskop microscope (Carl Zeiss).

For quantification of colocalization of cells expressing the Cy3-OMP and FITC-hormone, ImageJ software was used to calculate Pearson’s coefficient, based on the correlation between Red and Green signal overlap in at least three different normal human pituitary sections.

### SiRNA transfection

Short interfering RNA (siRNA) targeting OMP (siOMP-GCA GUU CGA UCA CUG GAA CGU GGU U) was synthesized by Invitrogen, and transfected into cells using Lipofectamine RNAiMAX (Invitrogen).

### Cell-cycle analysis

For the cell-cycle assay, 4.0 × 10^5^ cells were fixed with ice-cold 70% ethanol for 1 h on ice. The cells were then centrifuged at 800 rpm for 5 min, followed by resuspension in 1 ml of PI Master Mix containing 40 μL of propidium iodide (Invitrogen), 10 μL of RNase A (Sigma-Aldrich), and 950 μL of PBS. After incubation at 37 °C for 30 min, DNA ploidy was analyzed by flow cytometry.

### Cell proliferation assay

MTS assays were performed using the CellTiter 96 Aqueous One Solution Cell Proliferation Assay kit (Promega). To measure cell proliferation, siCON- or siOMP-treated cells were seeded into 96-well plates (3000 cells/well). Then, cell proliferation was measured every 24 h for 5 days. Briefly, 20 μL of MTS labeling reagent was added to each well and incubated at 37 °C for 1 h. Absorbance was measured at 490 nm. The relative proliferation of siOMP-treated cells was normalized to that of siCON-treated cells after background subtraction.

### Animals

Male OMP−/− transgenic mice were obtained from the Jackson Laboratory (B6;129P2-Omp^tm3Mom^/MomJ). Wild-type (WT) littermates served as controls. Mice were maintained under controlled conditions (12:12-h light/dark cycle, 21 °C) with free access to laboratory chow and tap water. All animal experiments were performed according to the applicable Korean laws, reviewed and approved by the Institutional Animal Care and Use Committee (IACUC) of the Yonsei University Severance Hospital, Seoul, Korea (IACUC Approval No: 2015-0025), and carried out in accordance with the approved guidelines by the IACUC.

Mice were genotyped by PCR using genomic DNA isolated from cut tails. The presence or absence of OMP was determined by multiplex PCR using primers OMP137, OMP138, and OMP139 according to genotyping conditions provided by the Jackson Laboratory (https://www.jax.org/strain/006667).

### Fura-2 Ca^2+^ assay

Cells were grown to 100% confluence in 96-well plates and washed with HBSS. They were then incubated in HBSS containing 3 μM Fura-2-AM (Invitrogen) at 37 °C for 30 min. Cells were washed thrice and incubated in HBSS at room temperature for 20 min to allow dye de-esterification. Fluorescence was detected every 5 s with a Gen 5 Luminescence spectrometer (BioTek, Winooski, VT, USA) at 340 and 380 nm (excitation) and 510 nm (emission). TRH (Sigma-Aldrich, St. Louis, MO, USA) was prepared in HBSS immediately before use. Fluorescence values are reported as *F*/*F*_0_, which was calculated using the following formula: (Δ*F* = (340 nm)_f_/(380 nm)_f_ − (340 nm)_0_/(380 nm)_0_).

### ELISA for PRL

GH4 cells (1 × 10^6^) were seeded in a 6-well plate (SPL Life Sciences, Pocheon, Korea) and cultured for 18 h. After two washes in a saline solution, the cells were incubated in a fresh culture medium with saline or TRH for 30 min. The medium was transferred to Eppendorf tubes that were centrifuged for 3 min at 700 × *g*. A 100 μl aliquot of supernatant was collected to determine the PRL concentration in the medium (ng/ml) using a mouse/rat enzyme-linked immunosorbent assay (ELISA) kit (Calbiotech). OMP-WT and OMP-KO mouse plasma samples were collected and frozen until use.

### Patients and samples

Human prolactinomas were derived from the Yonsei Pituitary Tumor Center (Seoul, South Korea). Briefly, human prolactinomas (*n* = 3, three female) were obtained during transsphenoidal surgery as part of an ongoing accession of human pituitary tumors. Tumors were frozen in liquid nitrogen and stored at −80 ℃ until use. Clinicopathological parameters were retrospectively collected from our institution. Normal pituitary glands (*n* = 3, three female) were obtained from the National Forensic Service (Gangwon-do, South Korea). The study was approved by the Institutional Review Board of Yonsei University, and informed consent was obtained for all subjects (IRB number: 4-2011-0740).

### Statistical analysis

Statistical analyses were carried out using Prism software v.4.0.0 (GraphPad Inc., La Jolla, CA, USA). Each experiment was repeated at least thrice. Statistical significance was determined using one-way analysis of variance followed by post-hoc Tukey's analysis, and Student’s *t*-test to compare the means of two different groups. Significant differences are indicated with asterisks (*P < 0.05; **P < 0.01; ***P < 0.001).

## Results

### OMP expression in rat pituitary cell lines and tissue

**Fig. 1:**
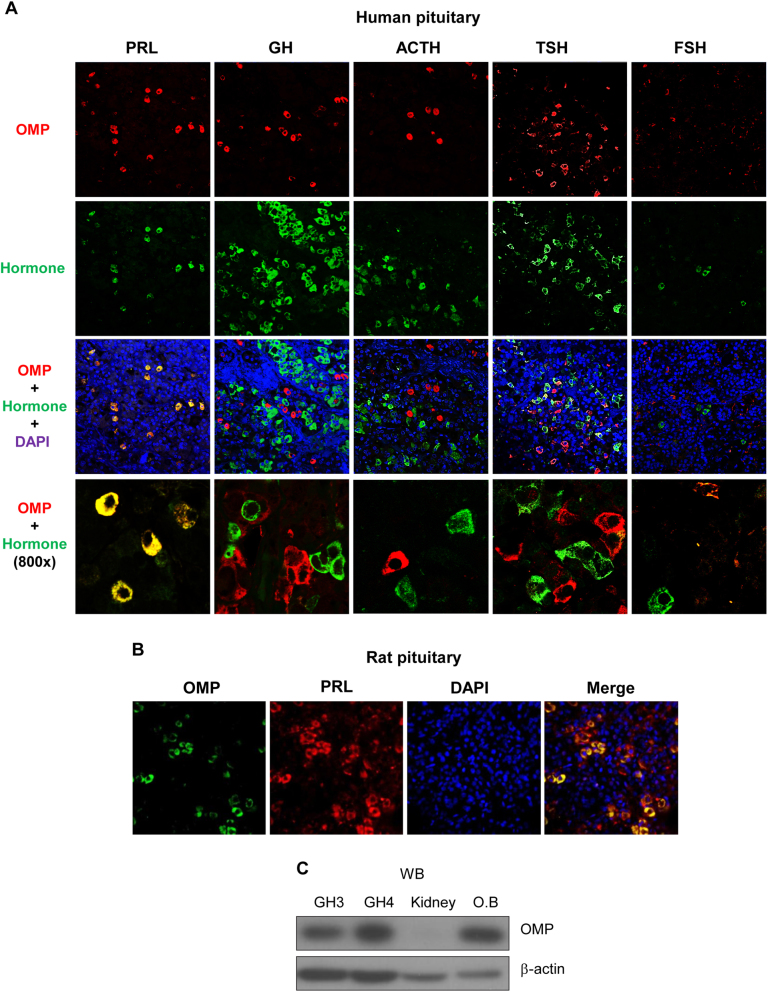
Expression of OMP in rat pituitary cell lines and tissue. **a** Representative images of OMP immunofluorescence labeling in normal human pituitary sections. OMP expression and all five cell types in the anterior pituitary were detected by antibodies against PRL, GH, ACTH, TSH, and FSH. OMP colocalized with PRL in lactotrophs, but not in other cell types. Images were obtained with a laser scanning microscope at 200× and 800× magnification. **b** Representative images of OMP and PRL immunofluorescence labeling in rat pituitary sections. **c** OMP expression in rat PRL-secreting cell lines. OMP protein levels in GH3 and GH4 cells, kidney (negative control), and olfactory bulb (positive control) was determined by western blotting. Blots are representative of three experiments

To determine whether OMP is expressed in the anterior pituitary gland, we carried out an immunohistochemical analysis using antibodies against various hormones, including PRL, growth hormone (GH), adrenocorticotropic hormone (ACTH), thyroid-stimulating hormone (TSH), and follicle-stimulating hormone (FSH) produced by somatotrophs, lactotrophs, adrenocorticotrophs, thyrotrophs, and gonadotrophs, respectively, of the human pituitary gland. We observed OMP-expressing cells significantly colocalized with PRL-expressing cells. In contrast, a portion of cells positive for TSH, FSH, GH, and ACTH was negative for OMP (Fig. 1a and S1 Fig). Consistent with these findings, rat pituitary immunolabeling revealed a high degree of PRL and OMP co-expression (Fig. 1b). We also examined OMP expression in GH3 and GH4 PRL-secreting rat pituitary cells by western blotting. OMP levels were found to be similar to those in the olfactory bulb (Fig. 1c), with a higher level observed in GH4 than in GH3 cells. These findings suggested that OMP plays an important role in pituitary lactotrophs.

### Loss of OMP expression increases PRL synthesis in vitro

**Fig. 2:**
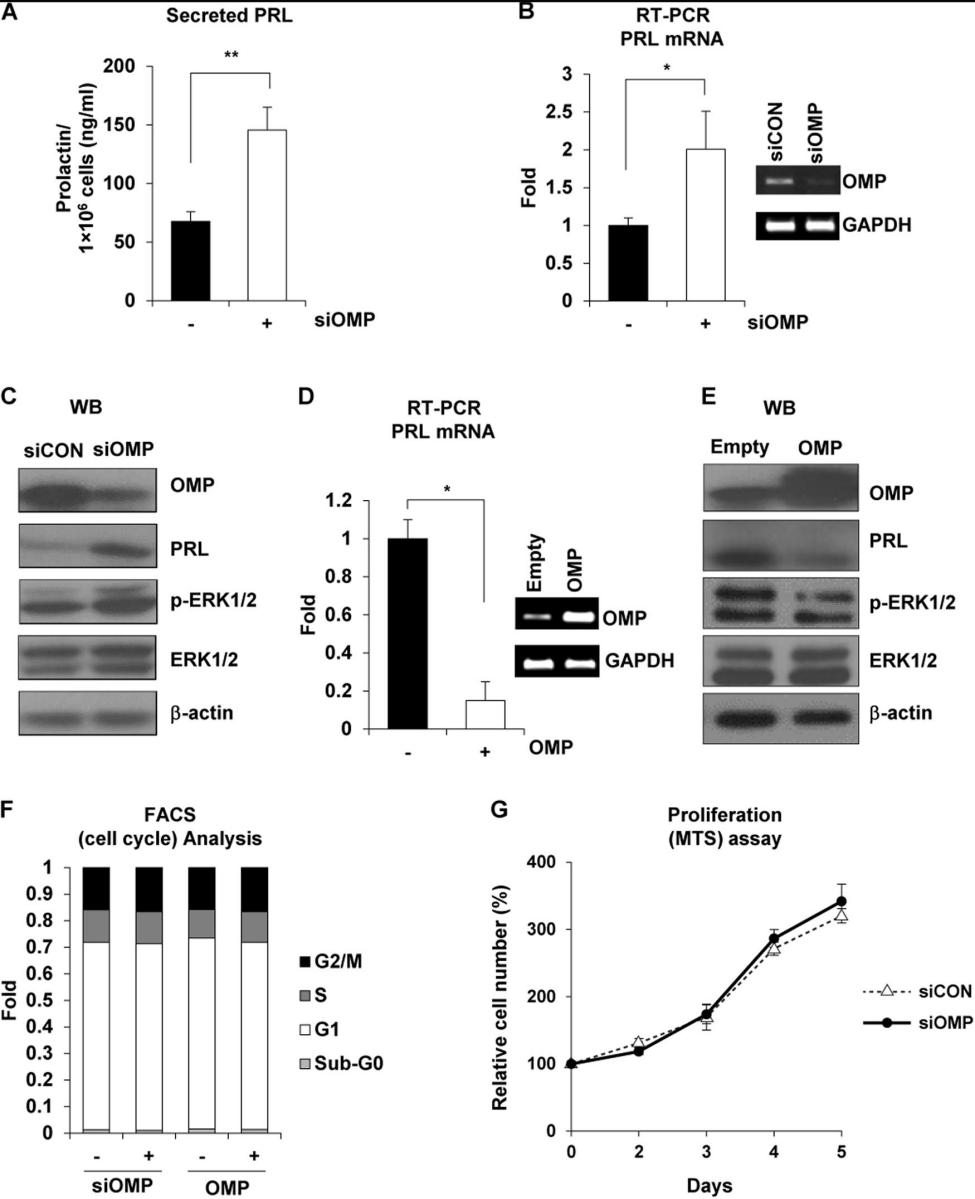
Effect of OMP deficiency on PRL synthesis in vitro. **a** PRL concentration in the supernatant of GH4 cells transfected with siCON or siOMP was evaluated using ELISA. Results represent the mean of at least three independent experiments. **P < 0.01 vs. siCON group. **b** RT-PCR analysis of PRL mRNA expression in GH4 cells transfected with siCON (−) or siOMP (+) (top). Semi-qRT-PCR analysis of OMP and glyceraldehyde-3-phosphate dehydrogenase (GAPDH) mRNA levels (bottom). *P < 0.05 vs. siCON group. **c** Effect of siCON or siOMP on the expression of indicated proteins in GH4 cells, as determined by western blotting. **d** RT-PCR analysis of PRL mRNA expression in GH4 cells transfected with empty vector (−) or OMP overexpression plasmid (+) (top). Semi-qRT-PCR analysis of OMP and GAPDH mRNA levels (bottom). Data represent mean ± SE of triplicate samples. *P < 0.05 vs. empty vector group. **e** Expression of indicated proteins in GH4 cells transfected with empty vector of OMP overexpression plasmid, as determined by western blotting. **f** GH4 cells were transfected with siCON or siOMP for 48 h, and cell-cycle distribution was determined by propidium iodide staining and fluorescence-activated cell sorting. **g** Proliferation of GH4 cells transfected with siCON or siOMP for 5 days. Data are representative of three experiments

As OMP was found to be expressed in human and rat lactotrophs, we investigated whether modulating its expression would affect PRL secretion. We used siRNAs to knock down OMP expression in GH4 cells and analyzed the levels of secreted PRL using ELISA. PRL levels were 2.15-fold higher in the culture supernatant of GH4 cells transfected with siOMP (siOMP-GH4 cells) than in control siRNA-transfected cells (siCON-GH4) (Fig. 2a). We examined the association between OMP expression and PRL synthesis using RT-PCR, and found that PRL mRNA levels were 2.01-fold higher in siOMP-GH4 than in siCON-GH4 cells (Fig. 2b). A similar trend was observed for PRL protein expression. Extracellular signal-regulated kinase (ERK) signaling is a point of convergence for PRL gene transcription^19^; a western blot analysis also revealed an increase in ERK1/2 phosphorylation in siOMP-GH4 cells, compared to that in the control cells (Fig. 2c and S2 Fig).

We also investigated whether OMP overexpression in lactotrophs could alter PRL gene expression. OMP-overexpressing GH4 (OMP-GH4) cells had 0.15-fold lower PRL mRNA levels than cells transfected with empty vectors (Empty-GH4) (Fig. 2d). Moreover, ERK1/2 phosphorylation was decreased in OMP-GH4 cells, compared to Empty-GH4 cells (Fig. 2e). As OMP knockdown increased ERK/12 phosphorylation, we investigated whether aberrant OMP-induced ERK1/2 activation could contribute to the development of pituitary hyperplasia or tumors. However, cell-cycle distribution and cell proliferation were unaltered in siOMP-GH4 cells (Fig. 2f, g). These data indicated that OMP inhibits PRL synthesis by suppressing ERK1/2 phosphorylation.

### OMP modulates TRH-induced ERK1/2 phosphorylation and Ca^2+^ influx

**Fig. 3:**
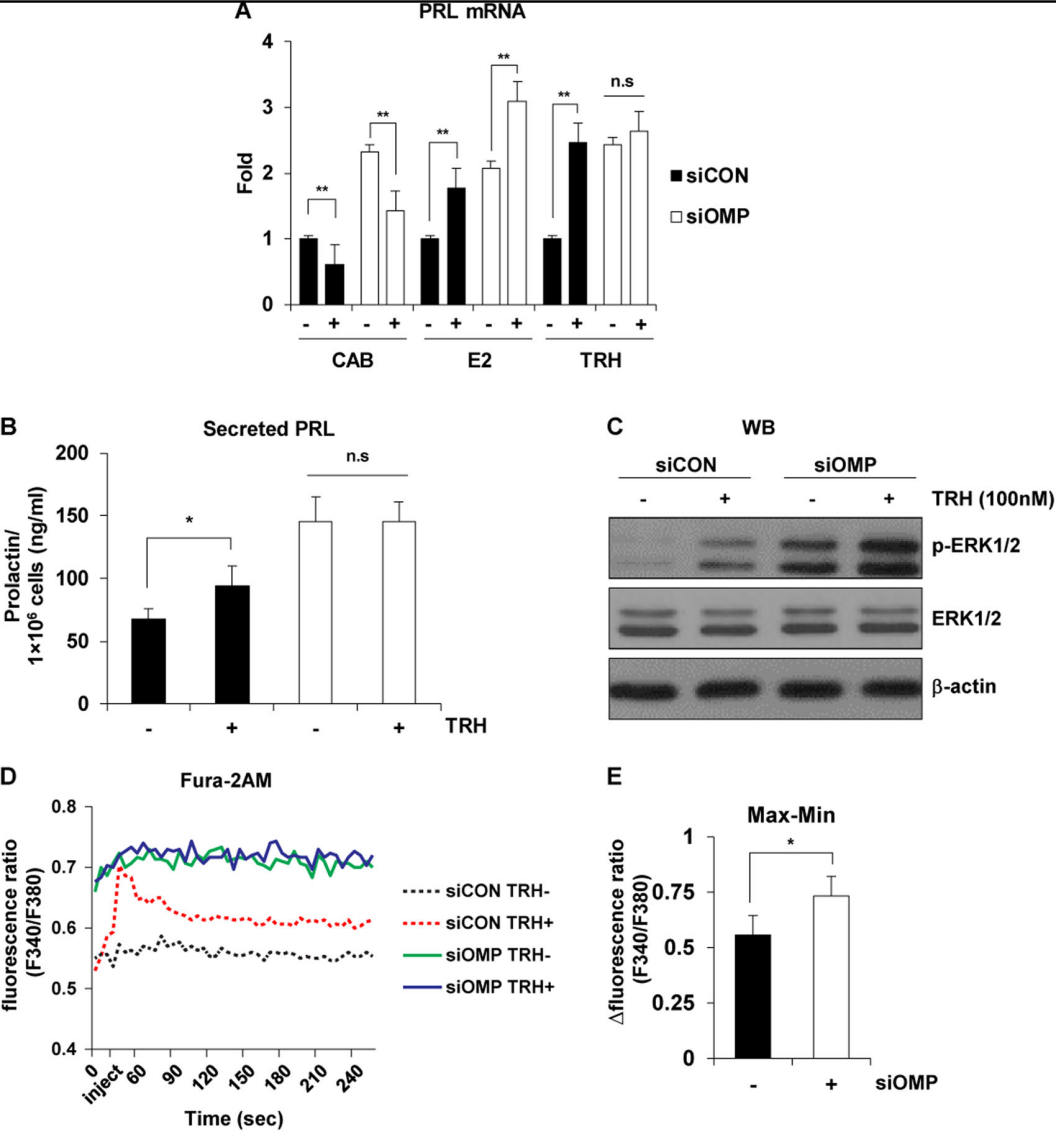
Role of OMP in TRH-induced ERK1/2 phosphorylation and Ca^2^^+^ influx. **a** RT-PCR analysis of PRL mRNA expression in GH4 cells transfected with siCON or siOMP and treated with Cab (1 μM), E2 (10 nM), or TRH (100 nM). Results represent the mean of at least three independent experiments. ns, not significant; **P < 0.01 vs. non-transfected (NT) control. **b** PRL secretion by GH4 cells transfected with siCON or siOMP. Results represent the mean of at least three independent experiments. ns, not significant; *P < 0.05 vs. NT control. **c** GH4 cells were transfected with indicated siRNAs and phosphate-buffered saline (PBS) or 100 nM TRH was added for 10 min, followed by western blotting. **d** GH4 cells were transfected with siCON or siOMP, then loaded with Fura 2-AM and treated with TRH (100 nM) to stimulate Ca^2+^ release. Intracellular Ca^2+^ levels were measured based on ratiometric measurements of absorbance at 340 and 380 nm (340/380). Intracellular Ca^2+^ levels were continuously monitored for 5 min. Values represent means. **e** Peak store-operated Ca^2+^ entry in PBS-treated siCON- and siOMP-transfected cells. Values represent mean ± SE. *P < 0.05

In pituitary lactotrophs, PRL gene expression is stimulated by neuropeptides such as TRH and E2, and is suppressed by dopamine via D2-type receptors^20–25^. Based on our finding that OMP could modulate PRL synthesis and secretion in pituitary lactotrophs, we investigated the signaling pathway involved. GH4 cells transfected with either siCON or siOMP were treated with the dopamine agonist cabergoline (Cab, 1 μM), β-estradiol (E2, 1 nM), or TRH (100 nM). There was no significant difference in PRL mRNA levels between siCON-GH4 and siOMP-GH4 cells following treatment with Cab or E2, as determined by real-time PCR (Fig. 3a). However, PRL expression was increased by 2.46-fold in siCON-GH4 cells by TRH treatment, whereas no change was observed in siOMP-GH4 cells (Fig. 3a). In addition, TRH treatment increased PRL secretion, whereas OMP knockdown abolished this effect (Fig. 3b). Moreover, ERK1/2 phosphorylation increased by about 4.82-fold in siCON-GH4 cells upon TRH treatment (Fig. 3c). ERK1/2 phosphorylation levels were similarly increased in siOMP-GH4 cells with or without TRH treatment (8.75- vs. 7.41-fold) (Fig. 3c and S3 Fig). These findings indicate that OMP inhibits TRH-induced ERK1/2 phosphorylation and PRL secretion.

In lactotrophs, TRH acts via a GPCR to increase phospholipase C activity, Ca^2+^ release from intracellular stores, and Ca^2+^ influx through L-type voltage-gated Ca^2+^ channels^26^. Based on these findings and the observations that basal PRL synthesis and secretion and TRH insensitivity were increased by blocking OMP expression in GH4 cells, we examined whether Ca^2+^ influx was also altered using the Fura-2-AM Ca^2+^ influx assay. Consistent with previous studies^26^, treatment with 100 nM TRH markedly increased intracellular Ca^2+^ levels in siCON-GH4 cells, but not in siOMP-GH4 cells (Fig. 3d). Moreover, basal intracellular Ca^2+^ levels were 1.33-fold higher in siOMP-GH4 cells than in siCON-GH4 cells (Fig. 3e). These data indicate that OMP regulates basal intracellular Ca^2+^ levels and TRH-induced PRL exocytosis.

### Loss of OMP expression leads to elevated basal PRL levels and loss of sensitivity to TRH in vivo

**Fig. 4:**
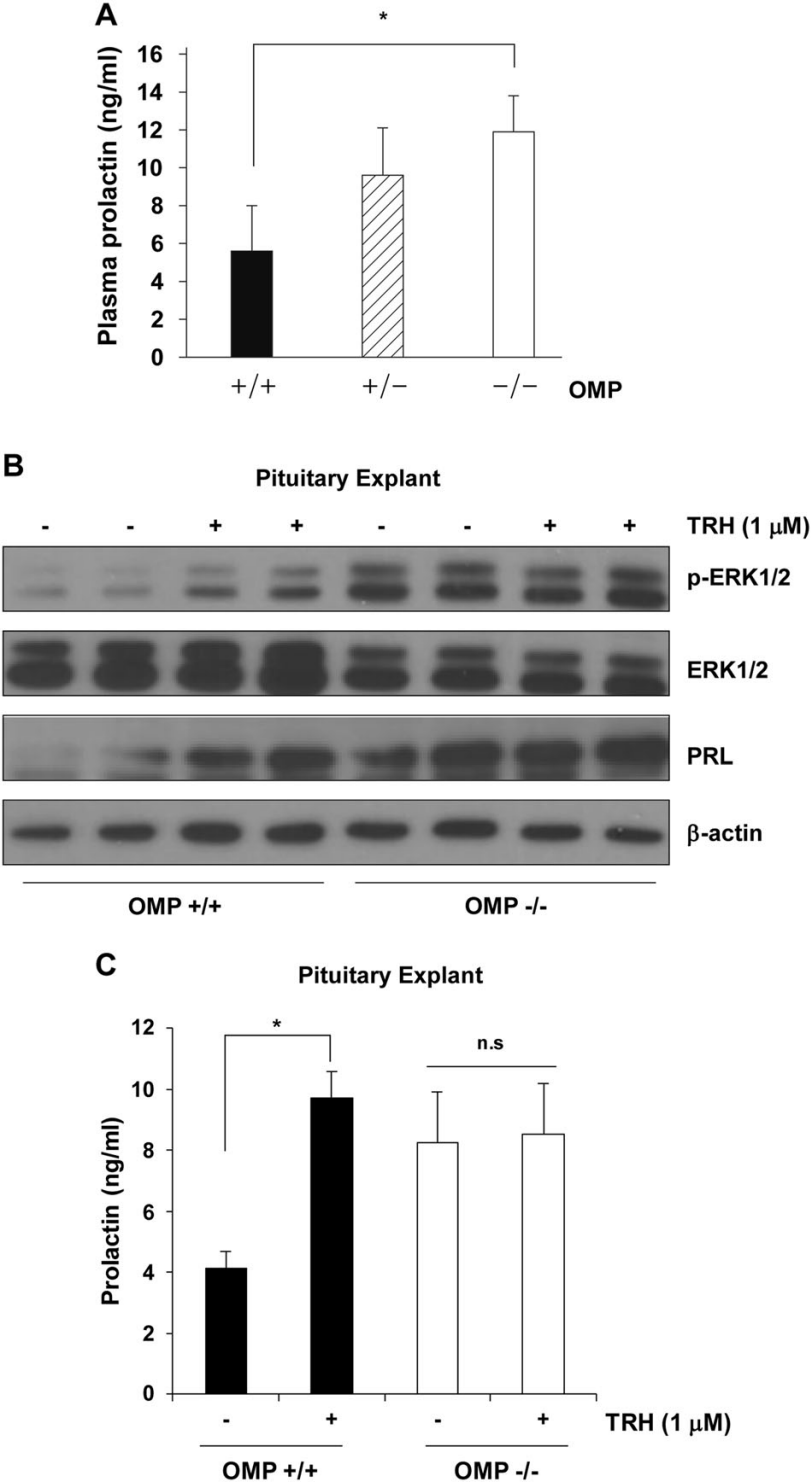
Loss of OMP expression increases basal PRL levels and leads to TRH desensitization in vivo. **a** Basal circulating PRL levels in OMP-WT (+/+), OMP-heterozygote (+/−), and OMP-KO (−/−) mice, as determined by ELISA. Values represent the mean ± SE (OMP+/+, *n* = 6; OMP+/−, *n* = 6; OMP−/− *n* = 6). *P < 0.05 vs. +/+ group. **b** ERK1/2 phosphorylation in OMP+/+ or OMP−/− anterior pituitary tissue treated with saline or TRH (1 μM), as determined by western blotting. β-Actin served as a loading control. **c** PRL levels in the culture supernatant of OMP+/+ and OMP−/− anterior pituitary tissue treated with saline or TRH (1 μM), as determined by ELISA. Results represent the mean of at least three independent experiments. ns, not significant; *P < 0.05 vs. (−) control

We next examined whether OMP expression in pituitary lactotrophs plays a role in basal PRL secretion and TRH sensitivity in vivo using OMP-knockout (OMP-KO) mice^6,27^. We evaluated circulating PRL levels in 20-week-old male OMP-WT (OMP-WT), OMP-heterozygote (OMP-het), and OMP-KO mice using ELISA, and found that basal circulating PRL levels were higher in OMP-KO than in OMP-WT mice (11.9 ± 2.3 ng/ml vs. 5.6 ± 0.6 ng/ml) (Fig. 4a).

To establish the function of OMP in anterior pituitary tissue, we examined ERK1/2 activation in primary tissue cultures. Consistent with the observed increase in circulating PRL concentration, OMP-KO mice showed a higher basal ERK1/2 phosphorylation level than OMP-WT mice. Anterior pituitary specimens from OMP-WT mice showed increased ERK1/2 phosphorylation upon TRH treatment, compared to those from saline-treated OMP-WT mice, whereas in OMP-KO mice, ERK1/2 phosphorylation levels were unchanged by TRH treatment (Fig. 4b).

To evaluate TRH-induced PRL secretion in the anterior pituitary, PRL levels in the supernatant of anterior pituitary tissue cultures were estimated using ELISA. Consistent with the results of the western blot, secreted PRL levels were 2.35-fold higher in cultures of OMP-WT anterior pituitary tissue treated with TRH than in those treated with saline (9.7 ± 0.9 vs. 4.13 ± 0.6 ng/ml). However, in the absence of OMP, secreted PRL levels were comparable between saline- and TRH-treated samples (8.24 ± 1.6 and 8.53 ± 1.7 ng/ml, respectively), indicating that the loss of OMP resulted in TRH desensitization (Fig. 4c). These data suggest that OMP mediates pituitary lactotroph PRL synthesis and TRH-induced PRL secretion.

### OMP expression is dysregulated in PRL-secreting pituitary adenoma

**Fig. 5:**
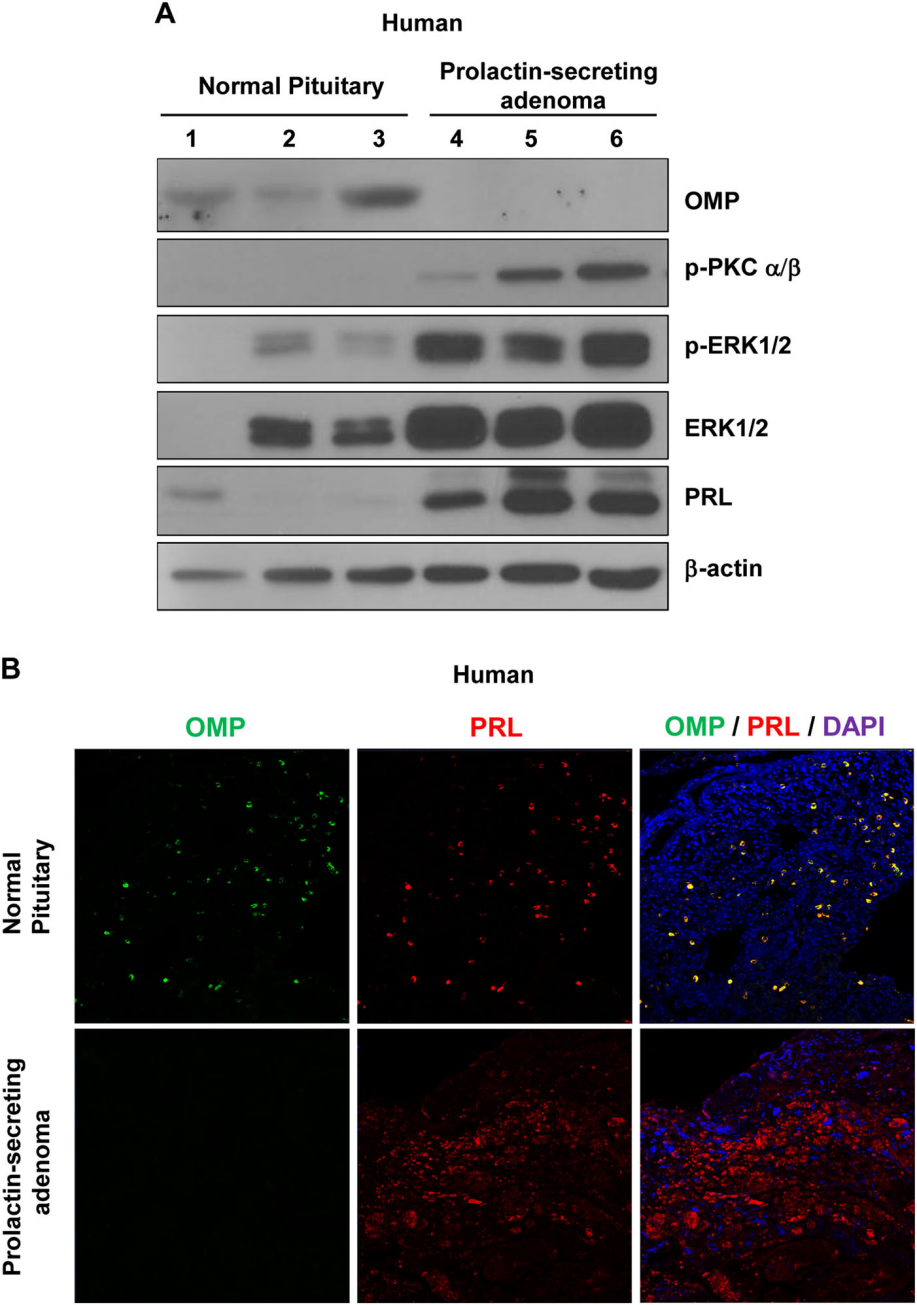
OMP expression in normal human pituitary and PRL-secreting pituitary adenoma. **a** Negative correlation between OMP and PKC-ERK1/2 levels in normal human pituitary (lanes 1–3) and prolactinoma (lanes 4–6) tissues, as determined by western blotting. β-Actin served as a loading control. **b** Representative images of OMP (green) and PRL (red) immunofluorescence labeling in normal and prolactinoma tissue sections. OMP colocalized with PRL in normal tissues, but was undetectable in prolactinoma. Images were obtained by laser scanning microscopy at 100× magnification

To confirm the regulation of PRL by OMP in the human pituitary, we analyzed OMP expression in normal pituitary and PRL-secreting adenoma (prolactinoma) tissue samples by western blotting. Consistent with results obtained in cell cultures and mice, protein kinase C (PKC) and ERK1/2 phosphorylation levels, as well as total ERK1/2 and PRL expression, were higher in prolactinoma than in normal pituitary tissues. In contrast, relative OMP expression was lower in the former than in the latter (Fig. 5a). An immunohistochemical analysis revealed that OMP was expressed in PRL-secreting cells of normal human pituitary tissue, but was almost undetectable in adenoma tissue. OMP expression was particularly low in areas of high PRL immunoreactivity (Fig. 5b). These results suggest that aberrant OMP expression in lactotrophs increase ERK1/2 activation and PRL secretion, thereby promoting prolactinoma and tumorigenesis.

## Discussion

The results of this study indicate that OMP is involved in PRL production and secretion by lactotrophs. OMP deficiency increased PRL expression and release in vitro and in vivo by failing to suppress ERK1/2 phosphorylation and modulate intracellular Ca^2+^ levels. In addition, OMP was found to be associated with PRL secretion induced by TRH, but not by other known neurohormones such as dopamine and E2. Finally, OMP expression was negatively correlated with PKC-ERK1/2 activation in human pituitary gland.

Recent studies have reported that OMP and ORs are expressed in non-olfactory tissues, including the pancreas, colon, bladder, and thyroid gland^15,18,28–30^. However, there have been no previous investigations on the expression and physiological functions of OMP in the pituitary gland. In the present study, we provide the first evidence that OMP is co-expressed with PRL in lactotrophs. Moreover, the higher expression of OMP in GH4 than in GH3 cells suggested its involvement in PRL regulation (Fig. 1), as GH4 cells are derived from GH3 cells and exhibit many features of lactotrophs^31–33^.

ERK1/2 activation plays an important role in the proliferation of PRL-secreting adenomas and in PRL secretion by the pituitary gland^34,35^. ERK1/2 phosphorylation was increased by OMP deficiency (Figs. 2, 4), which was associated with increased PRL secretion, but did not alter cell proliferation or induce tumorigenesis. This was consistent with a previous study, which demonstrated that the activation of ERK/mitogen-activated protein kinase (MAPK) in GH4 cells induced the differentiation of bihormonal somatolactotroph GH4 precursor cells into PRL-secreting lactotrophs. It was also reported that persistent activation of ERK/MAPK signaling not only failed to promote cell proliferation, but also reduced tumorigenesis of GH4 cells in vitro and in vivo^36^. These findings demonstrated that ERK1/2 activation induced by OMP deficiency was not associated with tumorigenesis, but promoted PRL production and secretion.

Pituitary PRL secretion is regulated by endocrine neurons in the hypothalamus. The most important of these are the neurosecretory tuberoinfundibulum neurons that secrete dopamine, which acts on lactotrophs to inhibit PRL secretion. In contrast, TRH stimulates PRL release. TRH receptor (TRHR) activation induces PKC, phosphatidylinositol, and Ca^2+^ signaling pathways^37,38^. We observed that Cab and E2 treatment similarly inhibited and stimulated PRL production in GH4 cells, respectively, irrespective of the presence or absence of OMP. However, TRH did not induce an increase in intracellular Ca^2+^ and ERK1/2 phosphorylation in cells and mice lacking OMP (Fig. 3c, e). We speculated that OMP modulated Ca^2+^-mediated PKC/MAPK signaling, which is the main pathway responsible for TRH-induced PRL expression. Previous studies have shown that OMP modulates Ca^2+^ extrusion from olfactory sensory neurons by directly interacting with brain-expressed X-linked 1 (Bex1) proteins, which can also bind to the Ca^2+^-dependent modulator, calmodulin (CaM)^8,11,13,39,40^. The OMP–Bex1–CaM interaction can explain the desensitization to TRH stimulation that is observed in the absence of OMP. Moreover, it has been reported that the Ca^2+^-CaM complex itself modulates PRL gene expression. These findings indicate that OMP plays a role in PRL production and secretion in lactotrophs through the modulation of Ca^2+^ and TRH signaling.

The observed desensitization might also have been due to the downregulation of TRHR in lactotrophs in the absence of OMP, as reduction in receptor density is an important mechanism for modulating cell responsiveness^41,42^. Interestingly, TRHR expression was decreased in GH4 cells and mice lacking OMP (S4 Fig). However, it is still unclear whether this effect is due to decreased transcription or increased mRNA degradation.

PRL plays a critical role in reproductive function; hyperprolactinemia is associated with anovulation and may directly or indirectly cause infertility^43^. We found that circulating PRL levels were increased in mice lacking OMP (Fig. 4a), which could explain the sub-fertile phenotype of these mice (https://www.jax.org/strain/006667). Interestingly, OMP was exclusively expressed in the normal pituitary gland, and not in prolactinoma tissues. Moreover, there was a negative correlation between PKC-ERK1/2 activation and low OMP expression (Fig. 5a and S5 Fig). The regulation of OMP by PKC-ERK1/2 is supported by reports that OMP can modulate olfactory signaling via Ca^2+^ extrusion^6,8,11,13,44^. We proposed that the absence of OMP in lactotrophs caused abnormalities in PRL synthesis and secretion.

In summary, this study provides evidence that OMP in lactotrophs of the anterior pituitary gland plays a role in normal production and secretion of PRL, and that associated molecules function in TRH-induced PRL secretion. These findings can aid in the development of improved strategies for managing hyperprolactinemia.

## Electronic supplementary material

Supplementary Figure (1-5)
