# Supplementary material for: Olfactory marker protein regulates prolactin secretion and production by modulating Ca2+ and TRH signaling in lactotrophs
Source: Exp Mol Med. 2018 Apr 6;50(4):15. doi: 10.1038/s12276-018-0035-z (PMC5938008; doi:10.1038/s12276-018-0035-z)
Supplement: Supplementary file 1 — Supplementary Figure (1-5) [file 12276_2018_35_MOESM1_ESM.doc]

**Olfactory marker protein regulates prolactin secretion and production by modulating Ca^2+^ and TRH signaling in lactotrophs**

Chan Woo Kang^1,2^, Ye Eon Han^1,2^, Mi Kyung Lee^3^, Jae Hyung Koo^4^, NaNa Kang^4^, Cheol Ryong Ku^2^, Eun Jig Lee^1,2^

**Running title:** OMP regulates prolactin secretion

**Author affiliations:**

^1^Brain Korea 21 PLUS Project for Medical Science, Yonsei University, Seoul, Korea

^2^Endocrinology, Institute of Endocrine Research, Yonsei University College of Medicine, Seoul, Korea

^3^Department of Pathology, NHIS Ilsan Hospital, 100 Ilsan-ro Ilsan-donggu, Goyang-si, Gyeonggi-do 10444, Korea

^4^Department of New Biology, DGIST, Daegu, Korea

**Corresponding authors**

Eun Jig Lee, MD, PhD

EJLEE423@yuhs.ac

Cheol Ryong Ku, MD, PhD

CR079@yuhs.ac


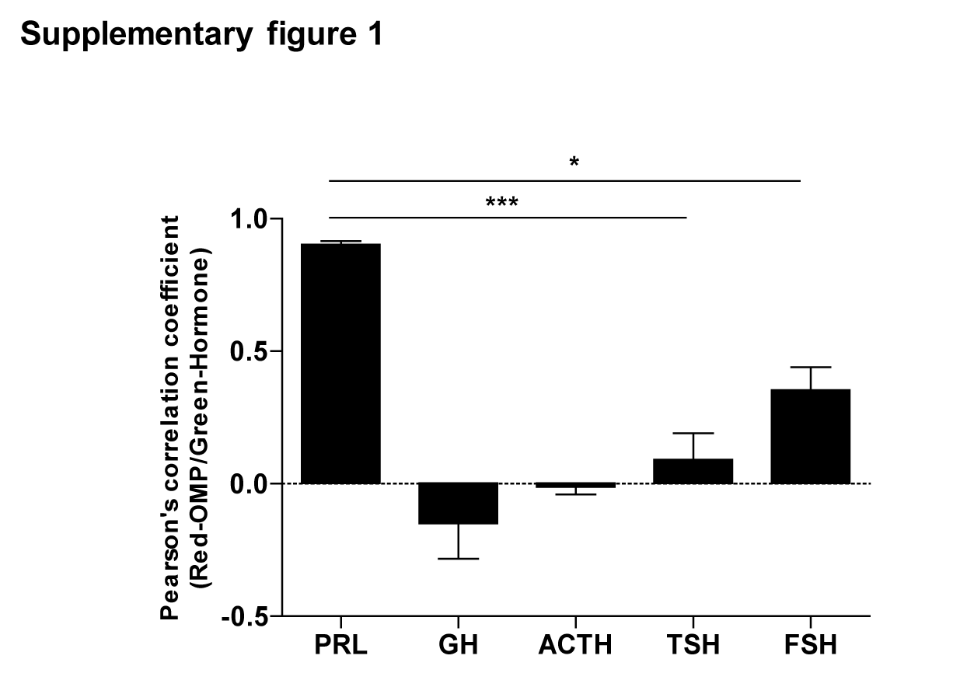


**Supplementary Figure 1**

Colocalization analysis of OMP and pituitary hormones. Pearson’s correlation coefficients (PCC) of images of the Cy3-OMP and FITC-PRL, FITC-GH, FITC-ACTH, FITC-TSH, or FITC-FSH in normal pituitary sections. Statistical analysis was carried out by the ANOVA followed by post-hoc Tukey analysis. ***, *P* < 0.001 PRL vs GH, ACTH, or TSH, *, *P* < 0.05, PRL vs FSH.


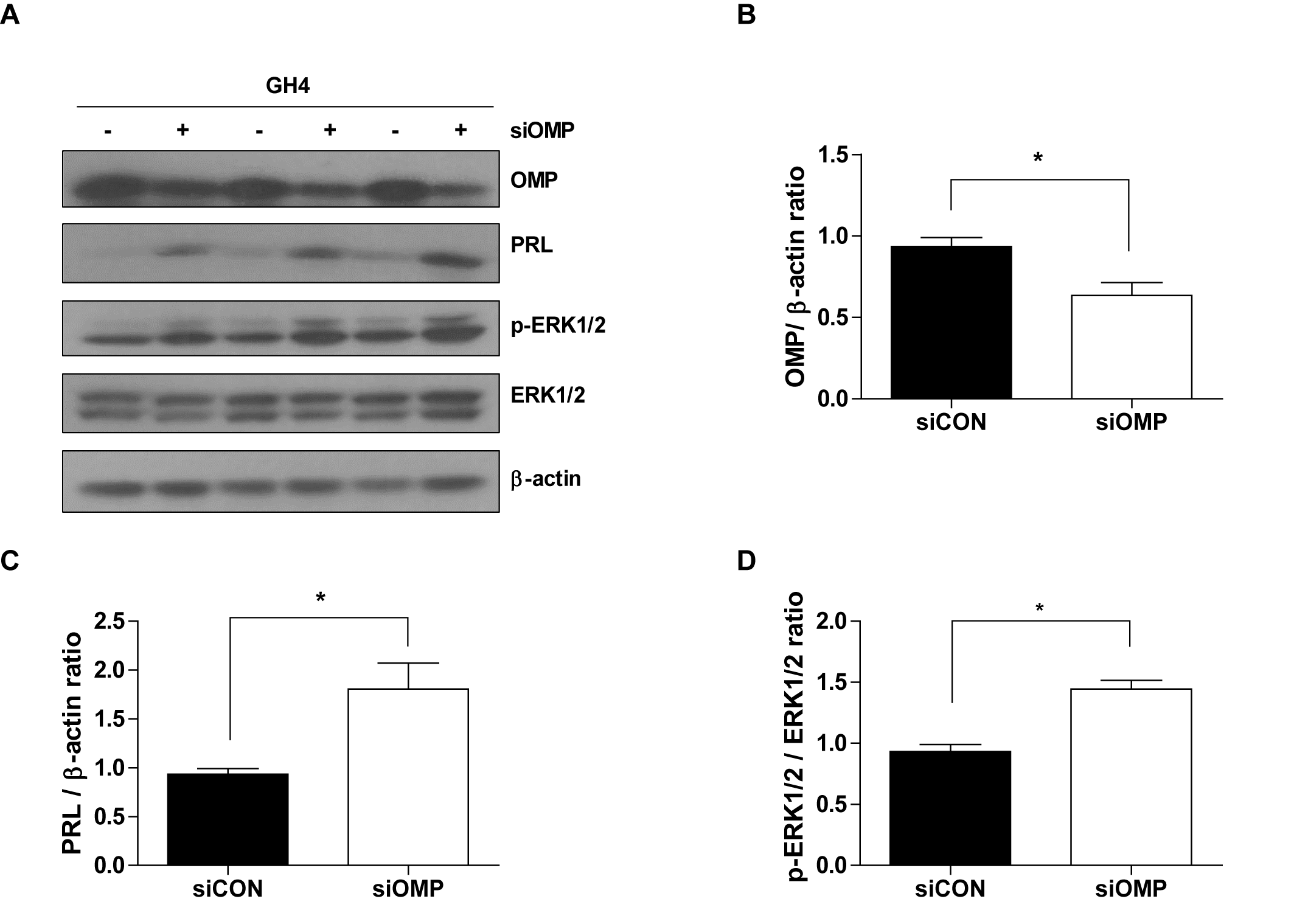


**Supplementary Figure 2**

**A**, Effect of siCON, or siOMP on the expression of indicated proteins in GH4 cells, as determined by western blot analysis.

**B**, Quantification of OMP, and **C**, PRL in siCON, or siOMP treated GH4 cells. Protein expression was quantified by densitometric analysis using image J software, normalized to β-actin expression, and graphed as a fold for each group. Data are presented as the mean as the mean ± SE of triplicate samples. *, *P* < 0.05 siCON-GH4 vs siOMP-GH4.

**D**, Quantification of p-ERK1/2 expression in siCON, or siOMP treated GH4 cells. p-ERK1/2 protein expression was quantified by densitometric analysis, normalized to total ERK1/2 expression, and graphed as a fold for each group. Data are presented as the mean as the mean ± SE of triplicate samples. *, *P* < 0.05 siCON-GH4 vs siOMP-GH4.


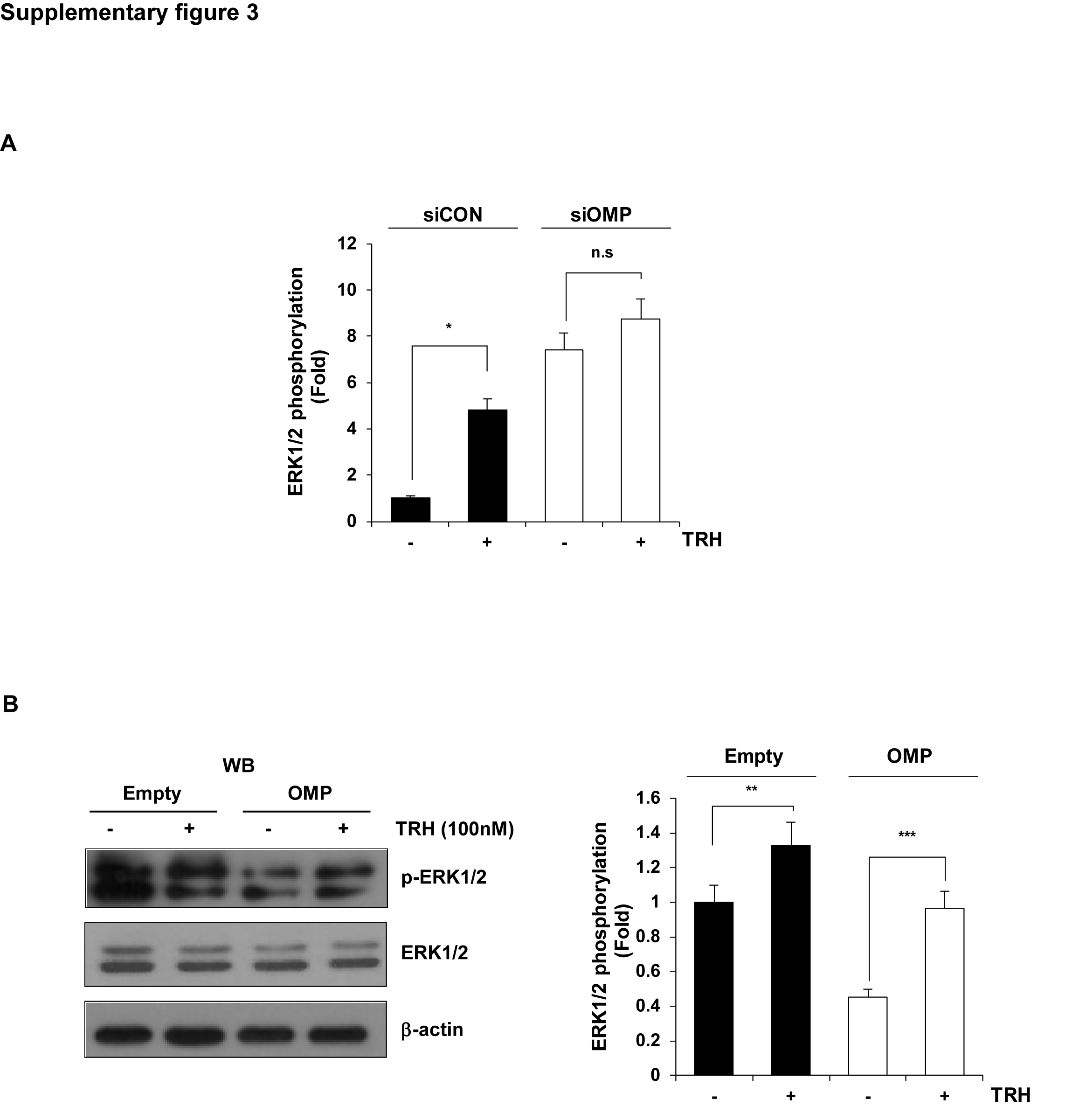


**Supplementary Figure 3**

**A**, Quantification of phosphorylated ERK1/2 expression in ± 100nM TRH siCON-GH4 and siOMP-GH4. Phosphorylated ERK1/2 protein expression from figure 3C was quantified by densitometric analysis using image J software, normalized to total ERK1/2 expression, and graphed as a fold for each group. Data are presented as the mean ± SE of triplicate samples. *, *P* < 0.05 vs siCON NT. n.s, not significant vs siOMP NT.

**B**, (Left) GH4 cells were transfected with Empty, or OMP-overexpressing plasmid. Then PBS or 100 nM TRH was added to each sample for 10 minutes and western blot analysis was performed. (Right) Quantification of phosphorylated ERK1/2 expression in ± 100nM TRH Empty transfected GH4 and OMP-expressing plasmid transfected GH4. Phosphorylated ERK1/2 protein expression from left panel was quantified, normalized to total ERK1/2 expression, and graphed as a fold for each group. **, *P* < 0.01 vs Empty NT. ***, *P* < 0.001 vs OMP-overexpressing plasmid NT.


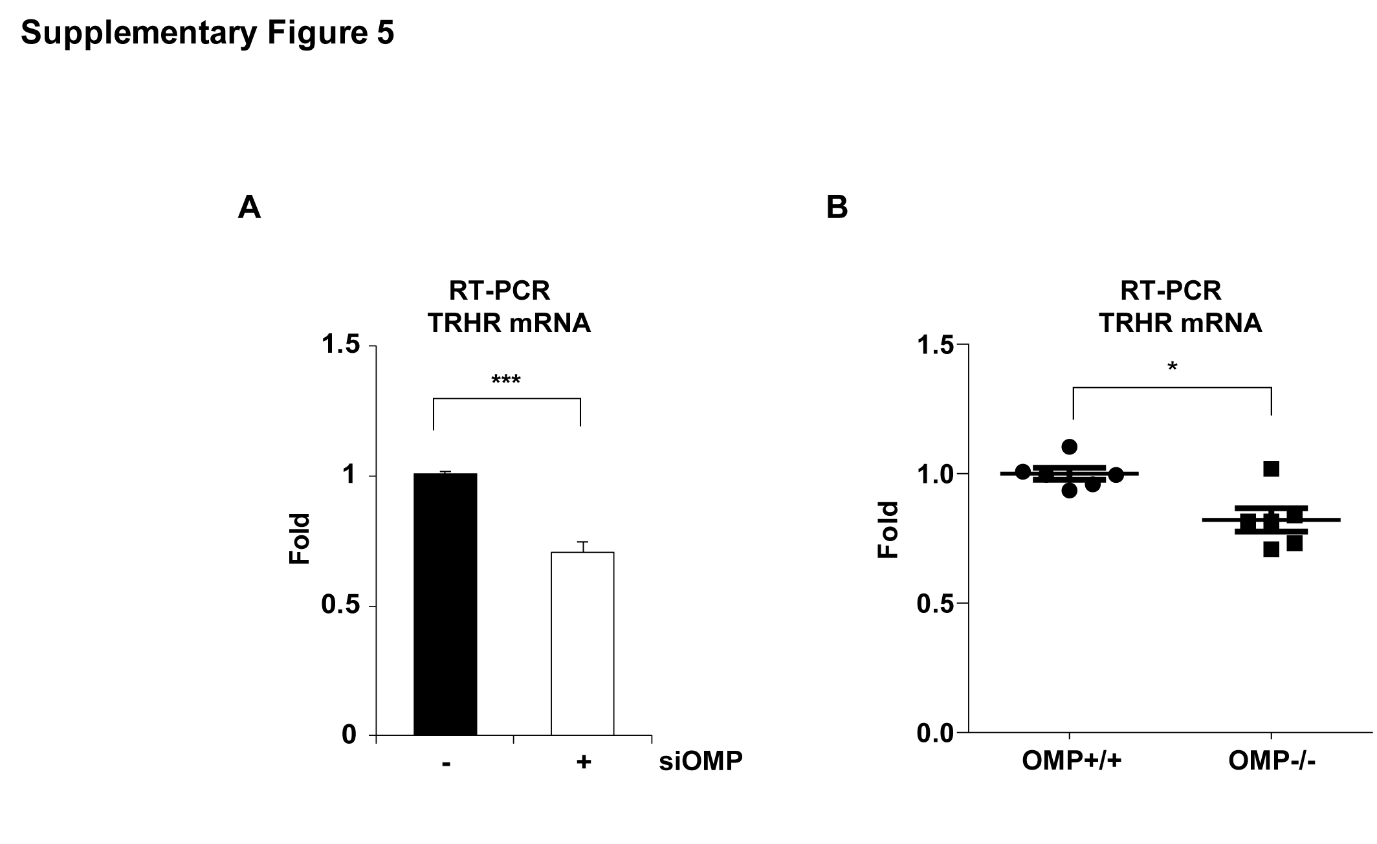


**Supplementary Figure 4**

**A**, RT-PCR analysis of thyrotropin-releasing hormone receptor (TRHR) mRNA expression in GH4 cells transfected with siCON or siOMP. Results represent the mean of at least three independent experiments. ***, *P* < 0.001 vs. siCON-GH4.

**B**, RT-PCR analysis of TRHR mRNA expression in olfactory marker protein (OMP)+/+ and OMP−/− mice. Results represent the mean of at least three independent experiments. *, *P* < 0.05 vs. OMP+/+.


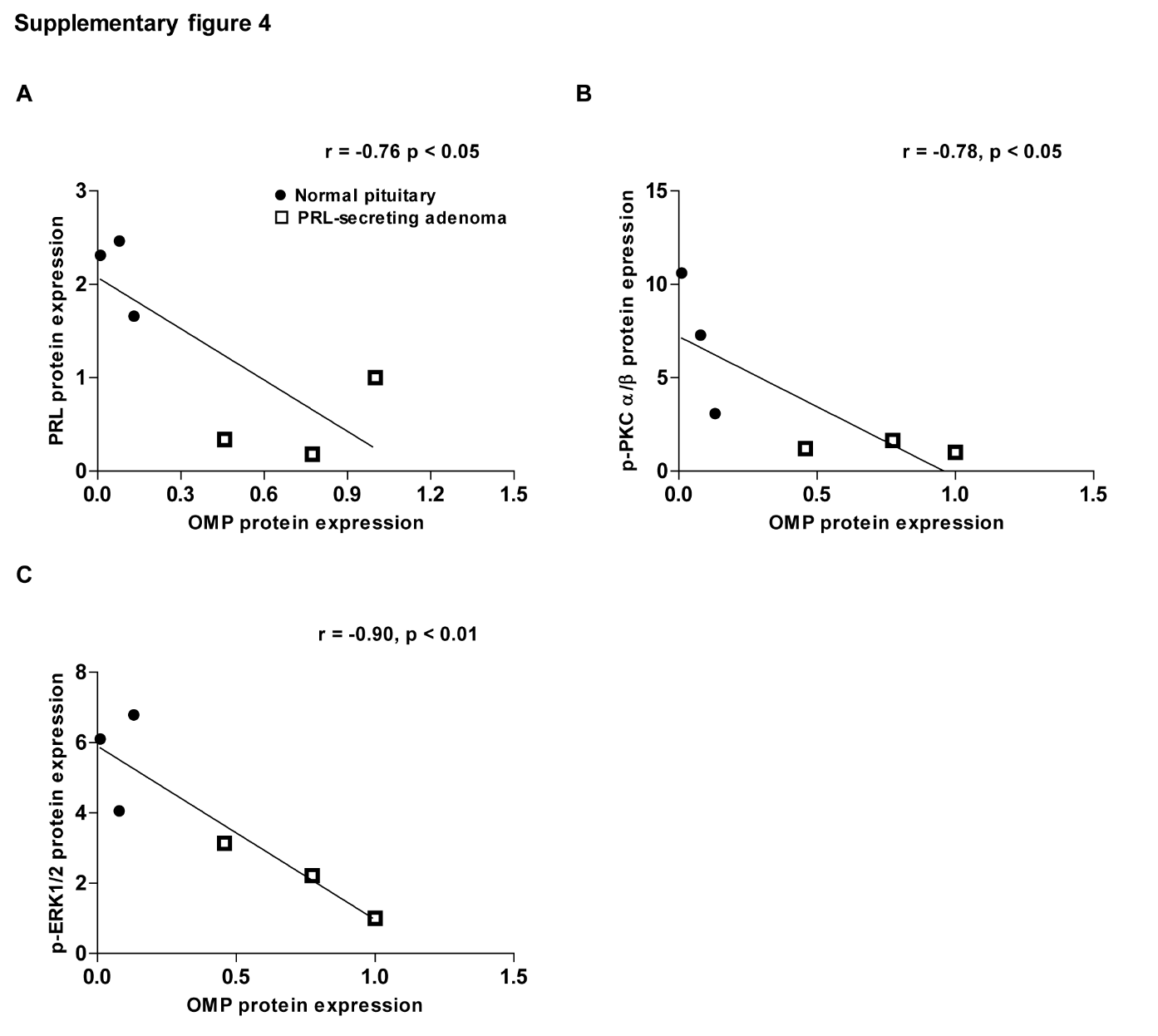


**Supplementary Figure 5**

OMP expression is dysregulated in PRL-secreting pituitary adenoma. Western blot analysis in figure 5A was analyzed by Pearson’s correlation coefficients and their statistical significance are indicated in panels.

**A**, Negative correlation between OMP protein expression levels and PRL protein expression (Pearson r = -0.76, *P* < 0.05).

**B,** Negative correlation between OMP protein expression levels and p-PKC α/β protein expression (Pearson r = -0.78, *P* < 0.05).

**C**, Negative correlation between OMP protein expression levels and p-ERK1/2 protein expression (Pearson r = -0.90, *P* < 0.01).
